# Examining New Mothers’ Access to Zuranolone

**DOI:** 10.31486/toj.25.0120

**Published:** 2026

**Authors:** Turner J. O. Simmers, David W. Galarneau

**Affiliations:** ^1^ The University of Queensland Medical School, Ochsner Clinical School, New Orleans, LA; ^2^ Department of Psychiatry, Ochsner Clinic Foundation, New Orleans, LA

In 2023, zuranolone became the first US Food and Drug Administration (FDA)-approved oral medication specifically for postpartum depression.^1^ Zuranolone is a novel neuroactive steroid that functions as a positive allosteric modulator of GABA_A_ receptors.^2^ Recommended to be taken once nightly for 14 days, zuranolone has a straightforward administration regimen^2^ and has been shown to be effective at providing rapid relief, with significantly decreased Hamilton Depression Rating Scale scores seen in patients at days 15 and 42 of treatment.^3^ Despite these advantages, many new mothers have faced barriers to accessing the drug, including high out-of-pocket costs and limited insurance coverage.^4^

Postpartum depression, a subset of the broader perinatal depression, is defined by the American College of Obstetricians and Gynecologists as a depressive mood disorder occurring within 12 months after childbirth.^5,6^ Consistent with this definition, the *Diagnostic and Statistical Manual of Mental Disorders, 5th Edition*, states that an estimated 7% of patients experience a major depressive episode between birth and 12 months postpartum.^7^ In addition, individuals with postpartum depression face an elevated risk of recurrent depressive episodes during and after subsequent pregnancies. Bloch et al reported that 42.5% of patients with postpartum depression experienced at least 1 episode of depression within 5 years, while 37.2% of patients had a recurrence of postpartum depression after their initial episode.^8^

In a Brazilian study of 706 mothers, those with postpartum depression had 6.5-fold higher odds of postpartum suicide risk compared to those without a mood disorder.^9^ Beyond maternal health, diminished socioemotional, fine motor, and language development has been documented in infants of mothers with postpartum depression.^10^ Burgeoning evidence further emphasizes the central role of maternal mental health in defining early relational and developmental outcomes. A study investigating birth trauma, postpartum depression, sleep disturbances, perceived social support, and relationship satisfaction found depressive symptoms to be the strongest negative predictor of mother-infant bonding.^11^ Similarly, in a cohort study of 83,109 mothers, maternal postpartum depression was associated with impaired mother-infant bonding at 1 year postpartum, even after controlling for 20 covariates.^12^ The combined maternal and infant risks underscore the pressing need for accessible and effective treatments.

The standard nonpharmacological treatment for postpartum depression is psychotherapy. Cognitive behavioral therapy and interpersonal therapy are frequently used psychotherapeutic modalities.^13^ Some patients experience symptom relief with psychotherapy alone, but as many as 76% of patients with postpartum depression will use pharmaceutical management during their care.^14^ Medication options are limited, particularly for mothers who are pregnant or breastfeeding at symptom onset. Selective serotonin reuptake inhibitors (SSRIs) are typically the first pharmacologic choice, with sertraline favored as little of the drug passes into breast milk, and extensive safety data are available.^15,16^ An alternative SSRI or a selective norepinephrine reuptake inhibitor can be used if the medication has previously been effective for the patient or if the mother does not tolerate sertraline. These alternatives require increased surveillance for adverse effects and infant safety because of the limited safety data.^16,17^ Patients commonly maintain their antidepressant regimen for 6 to 12 months to lessen the risk of relapse, with most patients experiencing symptom relief within 12 weeks.^17,18^

For patients seeking more rapid relief, pharmacologic options are limited. When it was available, brexanolone, an intravenous form of the progesterone metabolite allopregnanolone, provided meaningful relief of depressive symptoms within hours of administration. However, brexanolone therapy required a 60-hour continuous intravenous infusion that had to be performed in an inpatient setting because of potential serious side effects. The administration requirements, together with a high cost, ultimately led to the discontinuation of brexanolone.^4,19^

As a result, zuranolone is now the only available targeted therapy for postpartum depression, but patient access to zuranolone after FDA approval was limited by logistical and clinical barriers. Insurance coverage was a major initial barrier to access, as many insurers did not provide coverage for zuranolone, and even patients who were covered often faced complicated processes to gain access.^4^ For example, alternative treatments were required before certain patients receiving state-funded Medicaid could be eligible for zuranolone coverage, a requirement that could result in delays of more than a month.^4^ The drug's extremely high wholesale price of $15,900 further limited access for individuals without insurance.^20^ Access was further constrained by limited distribution, as zuranolone is not available through retail pharmacies but is dispensed only through a specialty pharmacy distribution network.^21^

Zuranolone also faced initial safety-related concerns because of limited lactation safety data, an important consideration for breastfeeding mothers.^22^ Data concerning the transfer of zuranolone into breast milk remain limited, although preliminary findings are reassuring. A 2024 phase 1 open-label study by Deligiannidis et al found that the relative infant dose of zuranolone was <1% in mothers who received a 14-day, once-daily 50-mg dose.^23^ By comparison, the generally acceptable relative infant dose threshold for alternative antidepressants is <10%, placing zuranolone comfortably within the threshold.^23^

As with many newly approved therapies, lack of prescriber familiarity with zuranolone may have influenced early adoption. During early rollout, some providers expressed hesitancy because of uncertainty regarding insurance coverage and lactation safety.^24^ In the first 6 months following FDA approval, only 2% of postpartum depression cases were treated with zuranolone, likely reflecting provider unfamiliarity, cost, and lack of insurance coverage.^25^ Efforts to improve provider comfort are currently underway, including the development of clinician-facing educational resources such as a University of North Carolina School of Medicine prescribing toolkit for clinicians.^26^ Furthermore, panel discussions and emerging research on potential additional indications for zuranolone suggest growing recognition by clinicians.^27,28^

For many patients, the challenges posed by the high cost and the lack of insurance coverage, provider familiarity, and safety data hindered therapy uptake, although financial data from 2025 suggest that access to zuranolone is improving.

In the second-quarter 2025 financial report, zuranolone manufacturer Sage Therapeutics, Inc, reported a 36% increase in prescriptions compared to the first quarter of 2025, with approximately 4,000 prescriptions dispensed to mothers with postpartum depression. The report also noted that more than 95% of insured women now had coverage or coverage provisions.^29^ These numbers represent a marked improvement from mid-April 2024, when only approximately 65% of insured patients had coverage.^30^

Despite expansions in insurance coverage, persistent barriers continue to delay access to zuranolone. Improving prescriber familiarity and expanding real-world data independent of manufacturer sources are strategies that could substantially enhance access. For instance, academic detailing (educational outreach) has been associated with increased prescriber uptake of targeted therapies, suggesting that similar clinician-focused education initiatives could encourage broader adoption of zuranolone.^31^ However, quantifying familiarity is difficult, as a paucity of literature examines prescribing trends or clinician perspectives, highlighting the need for expanded data to guide improvements. Reforming restrictive insurance policies, particularly prescription drug plan protocols, could improve patient access, as these policies have been shown to hinder patient access to psychiatric medications.^32^ Collectively, these strategies could help to ensure timely access to zuranolone for patients who need it, thereby maximizing the clinical and developmental benefits of prompt postpartum depression treatment.
